# Dosimetric comparison of TG‐43 formalism and HU‐based AcurosBV algorithms in HDR brachytherapy for head‐and‐neck superficial lesions with H.A.M. applicators

**DOI:** 10.1002/acm2.70554

**Published:** 2026-03-25

**Authors:** Pelei E. Kpohou, Lyu Huang, Lin Wang, Michael Trager, Yijian Cao

**Affiliations:** ^1^ Department of Physics and Astronomy Hofstra University Hempstead New York USA; ^2^ Northwell New Hyde Park New York USA; ^3^ Donald and Barbara Zucker School of Medicine at Hofstra/Northwell Hempstead New York USA; ^4^ Department of Radiation Medicine Northwell Health Lake Success New York USA

**Keywords:** AcurosBV, H.A.M. applicator, High‐dose‐rate brachytherapy, Model‐based dose calculation, surface applicator, TG‐43 formalism

## Abstract

**Background:**

TG‐43 formalism assumes homogeneous water media, potentially overlooking anatomical heterogeneities that affect dose accuracy. AcurosBV, a model‐based dose calculation algorithm, accounts for tissue and material heterogeneities. This study evaluates their dosimetric differences in high‐dose‐rate (HDR) brachytherapy using Harrison‐Anderson‐Mick (H.A.M.) applicators.

**Purpose:**

To compare dose‐volume histogram (DVH) metrics calculated using TG‐43 formalism and AcurosBV in patients treated with HDR brachytherapy using H.A.M. applicators for superficial lesions in the head and neck regions.

**Methods:**

Twenty patients (17 nasal, 3 non‐nasal) received HDR brachytherapy with H.A.M. applicators to a total dose of 40 Gy in eight fractions. Original plans calculated with TG‐43 formalism were retrospectively re‐calculated using AcurosBV (Eclipse v16.1), incorporating Hounsfield unit‐based heterogeneity corrections with dose‐to‐medium reporting. Planning target volumes (PTVs) were contoured by radiation oncologists; organs at risk (OARs) including brain, eyes, lens, optical nerve and skin were auto‐segmented using AutoContour (Radformation Inc., NY). DVH metrics (mean dose, D90%, V100%, V150%, V200%, D0.1cc, D0.2cc, D1cc and D2cc) were compared. The isodose distributions from both algorithms were also evaluated slice by slice. Statistical significance was assessed using a paired Student's *t*‐test (*p* < 0.05).

**Results:**

AcurosBV consistently reported lower DVH value in PTV than TG‐43. The average PTV mean dose was 48.65 ± 1.22 Gy (AcurosBV) versus 50.78 ± 1.40 Gy (TG‐43), with significant difference in V100 (−6.43 ± 0.95%, *p* < 0.003). OAR dose differences were modest (∼1.5%) except for the skin, which showed larger reductions (∼4–5% in D0.1cc–D2cc, *p* < 10^−^^8^). Visual inspection revealed less conformal isodose distributions in AcurosBV plans due to air cavities and tissue inhomogeneities.

**Conclusion:**

AcurosBV yielded lower and anatomically informed dose estimates compared with TG‐43 in HDR brachytherapy using H.A.M. applicators. These results support clinical adoption of model‐based algorithms but warrant caution, as dosimetric differences and prior TG‐43 experience may affect existing dose constraints and clinical outcomes.

## 1 INTRODUCTION

Brachytherapy is a widely used procedure in the management of various malignancies, offering the advantage of delivering high radiation doses directly to tumor sites while sparing adjacent healthy tissues.^1^, ^2^, ^3^, ^4^, ^5^ This is achieved by placing radioactive sources in close proximity to or within the tumor volume through intraluminal, intracavitary, interstitial or surface applicators. The radioactive source stays at its position over a specified period of time or permanently to deliver the planned doses. Brachytherapy can be used alone or in conjuncture with other radiation therapies. Compared to external beam radiation therapy, brachytherapy allows for rapid dose fall‐off, making it especially effective for treating superficial and anatomically complex targets.^6^

A critical component of brachytherapy planning is the accuracy of the dose calculation algorithms, which determine the amount of radiation delivered to the target and organs at risk (OAR). Inaccurate calculation results could cause underdosing in tumor or overdosing in normal tissues.^7^, ^8^ Historically, the American Association of Physicists in Medicine (AAPM) Taks Group 43 (TG‐43) introduced a semi‐empirical formalism in 1995, which has been the standard dose calculation algorithm in high‐dose‐rate (HDR) brachytherapy.^9^, ^10^ However, TG‐43 formalism assumes an infinite homogeneous water medium. This simplification facilitates the dose calculation but neglects the presence of tissue heterogeneities, applicator materials, or air cavities in which conditions for scattered radiation can be significantly altered in certain anatomical sites.^11^, ^12^, ^13^ This effectively treats the patient as a semi‐infinite medium with full‐scatter conditions and no external boundaries.

To address these limitations, model‐based dose calculation algorithms (MBDCAs) have been developed. Among these, AcurosBV, released by Varian Medical System Inc. in 2009, is a commercially available algorithm, which explicitly solves the linear Boltzmann Transport equation to calculate the radiation doses.^14^, ^15^, ^16^ It enables more accurate dose calculations by accounting for the tissue and material heterogeneities derived from the Hounsfield units in the patient's CT images. This algorithm has shown promising results in various clinical settings and is increasingly being considered for routine clinical use.^17^, ^18^, ^19^

Harrison‐Anderson‐Mick (H.A.M.) applicators are commonly used in HDR brachytherapy for treating skin, mucosa, and postoperative beds in anatomically complex regions of the head and neck.^20^, ^21^ Their flexible construction allows them to conform to curved surfaces such as the nose, cheek, and temple; however, air gaps between the applicator and skin, adjacent air cavities, and local bone can substantially alter scatter conditions. Under these circumstances, the homogeneous, unbounded water assumption of TG‐43 may lead to clinically relevant discrepancies compared with model‐based calculations. Although several studies have compared TG‐43 and model‐based algorithms in gynecologic, breast, and lung HDR brachytherapy, there are limited data specifically addressing H.A.M.‐based superficial treatments in the head‐and‐neck region. Accordingly, this work quantifies TG‐43 versus HU‐based AcurosBV differences in target coverage and OAR doses for a clinical cohort treated with H.A.M. applicators, and provides practical considerations for a safe clinical transition to model‐based dose calculation in this near‐surface setting.

## 2 METHODS

### 2.1 Patient selection

This retrospective study included 20 patients treated at a single institution between June 2022 and February 2024 with HDR brachytherapy using H.A.M. applicators for superficial lesions in the head and neck region. Seventeen patients were treated for nasal lesions, while the remaining three had lesions on the cheek, posterior neck, and right temple, respectively. All lesions were superficial skin or mucosal targets. All patients received a total prescribed dose of 40 Gy delivered in 8 fractions of 5 Gy each. Treatments were delivered according to institutional superficial brachytherapy protocols, typically twice a week. Plans were normalized such that the 100% isodose line corresponded to the prescription dose, encompassing a subcutaneous depth of 3–5 mm while maintaining organ‐at‐risk (OAR) constraints as determined by the radiation oncologists.

### 2.2 Imaging, treatment planning, and contouring

All patients underwent CT simulation in the treatment position using a Siemens SOMATOM Definition AS scanner. BBs or wires were placed to help guide target contouring during planning. Images were acquired at 120 kVp with automatic mA modulation, reconstructed at 2 mm slice thickness and a field of view 500 mm. The Harrison‐Anderson‐Mick (H.A.M.) applicators were positioned directly on the skin surface by the radiation oncologists and immobilized using tape and/or a thermoplastic mask per departmental practice to minimize air gaps and displacement during imaging and treatment.

Initial treatment plans were generated using the TG‐43 formalism in BrachyVision module of the Eclipse v16.1 (Varian Medical Systems Inc., Palo Alto, CA). For this study, each plan was retrospectively recalculated using the AcurosBV algorithm, which incorporates the Hounsfield unit (HU)‐based tissue inhomogeneity corrections and accounts for applicator materials. The dose is reported as dose to local medium.

Target volumes were delineated and approved by radiation oncologists. OARs, including the eyes, lens, optic nerves, etc., were first automatically segmented using the AutoContour scripts from Radformation Inc., and then reviewed and refined by medical physicists. Manual adjustments were made at the discretion of the reviewers to ensure anatomical accuracy before dose evaluation.

### 2.3 Dosimetric metrics for comparison

Dosimetric evaluation focused on the dose volume histogram (DVH) metrics for the planning target volume (PTV) and OARs. For the PTV, the following parameters were analyzed: mean dose, D90%, V100%, V150%, and V200%. For OARs, the evaluated DVH metrics included D0.1cc, D0.2cc, D1cc, and D2cc. Each patient`s plan was calculated with TG‐43 formalism and AcurosBV algorithm. All DVH data were collected using Eclipse for direct comparison. Summary statistics were compiled in Excel, and statistical testing and figure generation were performed in Python.

For each DVH metric, the difference between the two algorithms was defined as:

$$\Delta Metric\;=\;\mathrm{Metric}\left(\mathrm{AcurosBV}\right)\;-\;\mathrm{Metric}\left(\mathrm{TG}-43\right)$$

All statistical comparisons were performed on a per‐patient basis (*n* = 20), using one plan per patient that was recalculated with AcurosBV from the original TG‐43 plan. Statistical significance between TG‐43 and AcurosBV was assessed using a paired two‐tailed Student's t‐test with a significance threshold of *p* < 0.05. Formal normality testing of the paired differences was not performed. Box‐and‐whisker plots were generated to illustrate inter‐patient variability and the distribution of metric differences. Isodose distributions from both algorithms were reviewed on a slice‐by‐slice basis as a qualitative assessment of spatial discrepancies; no quantitative spatial conformity metric was calculated in this study. Unless otherwise stated, uncertainties reported in tables represent twice the standard error (approximately a 95% confidence interval for the population mean).

### 2.4 Artifact evaluation

To evaluate the potential influence of streaking artifacts and small high‐density objects on AcurosBV dose calculations, we performed a sensitivity analysis on a subset of three cases. These cases were selected because they exhibited visibly prominent CT artifacts or contained BB markers and dummy source wires within or near the PTV, representing worst‐case scenarios in our cohort. In each of these three cases, the artifacts, BBs and dummy source were contoured and their HUs overridden as water or air accordingly. AcurosBV dose was then recalculated and DVH metrics were compared with the corresponding values obtained from the original (non‐overridden) CT dataset. HU overrides were applied only for this sensitivity analysis; all primary study results (DVH summaries and statistical comparisons) are based on the original clinical images to maintain consistency across the cohort.

## 3 RESULTS

### 3.1 Difference in DVH metrics in PTV

**FIGURE 1:**
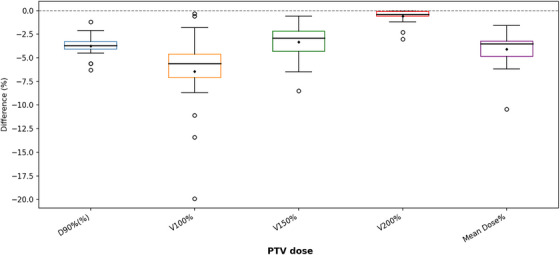
Box‐and‐whisker plot of DVH metrics in PTV illustrating the percentage differences between AcurosBV and TG‐43 calculations. The horizontal line at 0% denotes the reference dose level as calculated by the TG‐43 formalism.

Across all cases, dose metrics calculated with AcurosBV were consistently lower than those obtained with TG‐43 formalism. The average mean dose to the PTV was 48.65 ± 1.22 Gy with AcurosBV, compared to 50.78 ± 1.40 Gy with TG‐43, representing a mean absolute reduction of 2.13 Gy (approximately −4%). Similar trends were observed across other DVH metrics as shown in Table 1 and Figure 1. The D90%, representing the minimum dose received by 90% of the PTV, was 86.47 ± 2.97% with AcurosBV and 90.21 ± 3.07% with TG‐43. The V100%, which denotes the percentage of the PTV receiving 100% of the prescribed dose, showed the largest difference of approximately −6%. Reductions in V150% and V200% were also observed, with an average difference of −3% and −0.5% respectively. All differences in PTV DVH metrics were statistically significant, with p‐value less than 0.05.

**TABLE 1:** Summary of DVH metrics difference (mean ± SE, with 1st and 3rd quartiles (Q1–Q3)) in target volumes calculated by TG‐43 formalism and AcurosBV dose calculation algorithm (*n* = 20). Differences are reported as *DVH_Metric_AcurosBV—_DVH_Metric_TG‐43._*

DVH metrics	Median (Q1–Q3)	Mean ± SE	*p* value
Mean dose (Gy)	−3.53 (−4.95 to −3.23) (Gy)	−4.08 ± 0.39 (Gy)	<$10^{-6}$
D90 % (%)	−3.7 (−4.1 to −3.2)	−3.75 ± 0.25	<$10^{-11}$
V100 % (%)	−5.6 (−7.15 to −4.5)	−6.43 ± 0.95	<$10^{-5}$
V150 % (%)	−2.9 (−4.65 to −1.9)	−3.30 ± 0.43	<$10^{-6}$
V200 % (%)	−0.4 (−0.6 to −0.1)	−0.57 ± 0.17	<0.003

**FIGURE 2:**
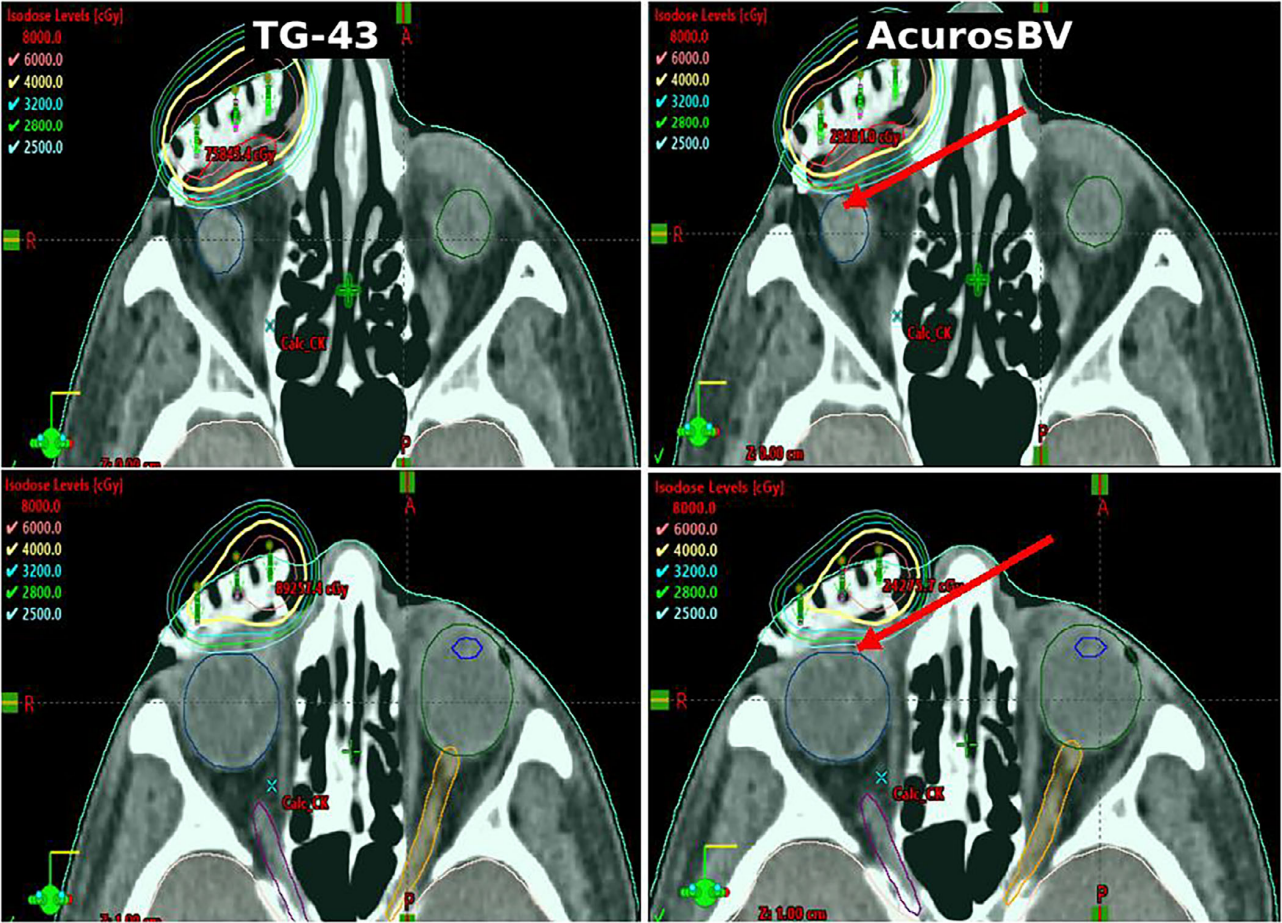
Comparison of isodose distributions between TG‐43 (left) and AcurosBV (right) within the treatment planning system. Arrows indicate representative cold‐spot regions adjacent to air cavities and/or bony interfaces.

The isodose distribution comparisons reinforced these findings. Visual analysis across axial slices demonstrated that dose distributions generated using AcurosBV appeared less conformal and cooler, particularly in regions adjacent to inhomogeneities (Figure 2). These visual observations correlated well with the observed numerical reductions in PTV DVH metrics and illustrate the effect of heterogeneity correction in anatomically complex region.

### 3.2 Difference in DVH metrics in OAR

**FIGURE 3:**
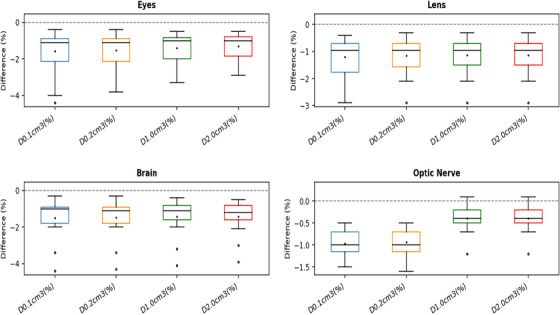
Box‐and‐whisker plot of DVH metrics in OARs illustrating the percentage differences between AcurosBV and TG‐43 calculations. The horizontal line at 0% denotes the reference dose level as calculated by the TG‐43 formalism.

The DVH metrics of OAR revealed generally minor differences between the two calculation methods as shown in Table 2 and Figure 3. For structures such as the brain, optic nerves, lenses, and eyes, the average dose difference remained within approximately 1.5%, with AcurosBV consistently predicting slightly lower values. One exception was noted in a single patient case where the optic nerve demonstrated a marginally higher dose with AcurosBV (up to 0.1%) for D0.1cc, and D0.2cc. Given the very small magnitude, this likely reflects local heterogeneity and finite‐scatter effects near bony/air interfaces rather than a clinically meaningful increase. Despite the small magnitude of these changes, all comparisons of OAR dose metrics between the two algorithms yielded statistical significance (*p* < 0.05), reflecting the consistency of the direction of change across patients.

**TABLE 2:** Summary of DVH metrics difference (mean ± SE, with 1st and 3rd quartiles (Q1–Q3)) in OARs calculated by TG‐43 formalism and AcurosBV dose calculation algorithm (*n* = 20). Differences are reported as *DVH_Metric_AcurosBV—_DVH_Metric_TG‐43_*.

OARs	DVH metrics	Median (Q1–Q3)	Mean ± SE	*p* value
	D0.1cc (%)	−1.1 (−2.2 to −0.9)	−1.57 ± 0.21	<$10^{-7}$
Eyes	D0.2cc (%)	−1.1 (−2.2 to −0.9)	−1.53 ± 0.18	<$10^{-8}$
	D1cc (%)	−1.0 (−2.0 to −0.8)	−1.40 ± 0.15	<$10^{-9}$
	D2cc (%)	−1.0 (−1.9 to −0.8)	−1.3 ± 0.12	<$10^{-10}$
	D0.1cc (%)	−1.0 (−1.8 to −0.7)	−1.20 ± 0.13	<$10^{-8}$
Lens	D0.2cc (%)	−1.0 (−1.6 to −0.7)	−1.16 ± 0.12	<$10^{-8}$
	D1cc (%)	−1.0 (−1.5 to −0.7)	−1.14 ± 0.12	<$10^{-9}$
	D2cc (%)	−1.0 (−1.5 to −0.7)	−1.14 ± 0.12	<$10^{-9}$
	D0.1cc (%)	−1.0 (−1.9 to −0.9)	−1.49 ± 0.25	<$10^{-4}$
Brain	D0.2cc (%)	−1.1 (−1.9 to −0.9)	−1.48 ± 0.25	<$10^{-4}$
	D1cc (%)	−1.1 (−1.7 to −0.8)	−1.42 ± 0.23	<$10^{-4}$
	D2cc (%)	−1.2 (−1.7 to −0.8)	−1.42 ± 0.21	<$10^{-5}$
	D0.1cc (%)	−1.0 (−1.2 to −0.7)	−0.97 ± 0.08	<$10^{-7}$
Optic_Nerve	D0.2cc (%)	−1.0 (−1.2 to −0.7)	−0.93 ± 0.08	<$10^{-7}$
	D1cc (%)	−0.4 (−0.5 to −0.2)	−0.39 ± 0.08	<0.0002
	D2cc (%)	−0.4 (−0.5 to −0.2)	−0.39 ± 0.08	<0.0002
	D0.1cc (%)	−5.0 (−6.7 to −3.9)	−5.54 ± 0.57	<$10^{-8}$
Skin	D0.2cc (%)	−5.0 (−6.0 to −4.1)	−5.14 ± 0.37	<$10^{-10}$
	D1cc (%)	−4.2 (−5.1 to −3.6)	−4.66 ± 0.36	<$10^{-10}$
	D2cc (%)	−4.0 (−4.9 to −3.2)	−4.35 ± 0.36	<$10^{-10}$

**FIGURE 4:**
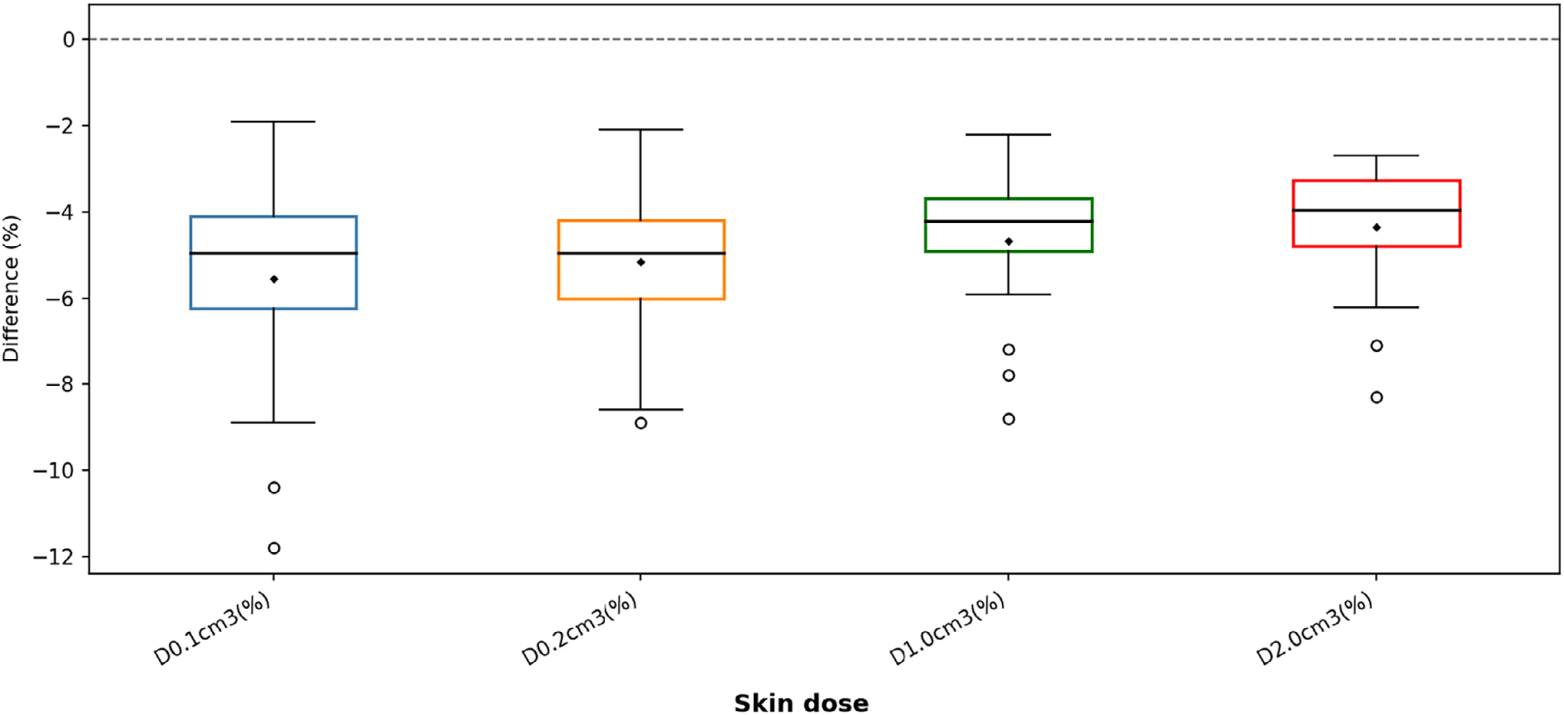
Box‐and‐whisker plot of DVH metrics in skin illustrating the percentage differences between AcurosBV and TG‐43 calculations. The horizontal line at 0% denotes the reference dose level as calculated by the TG‐43 formalism.

In contrast, Table 2 and Figure 4 showed more pronounced differences in the dose to the skin. AcurosBV reported consistently lower skin doses than TG‐43. The reductions were approximately 5% for D0.1cc and D0.2cc, and around 4% for D1cc and D2cc. These discrepancies were statistically significant (*p* < 0.05) and may be attributed to the geometry of the treated regions, particularly the nose, where achieving full contact between the H.A.M. applicator and the irregular skin surface is difficult, potentially resulting in air gaps and increasing dose uncertainty.

**FIGURE 5:**
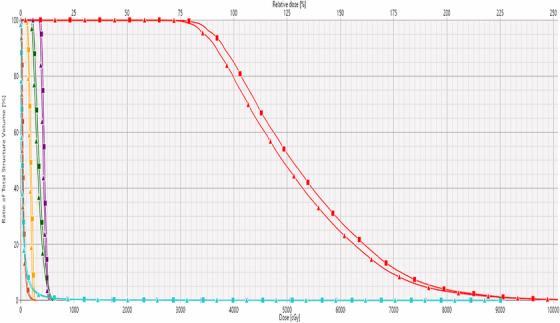
DVH comparison of the PTV and OARs between the AcurosBV algorithm and TG‐43 formalism within the treatment planning system. Structures are color‐coded as follows: brain (gray), skin (blue), optic nerve (orange), eye (green), lens (purple), and PTV (red).

Figure 5 illustrates an example of the cumulative DVHs between the AcurosBV and TG‐43 formalism for both the PTV and OARs. A dose reduction trend is observed with the AcurosBV calculation, as characterized by a leftward shift in DVH curves relative to those generated with TG‐43.

### 3.3 The effect of CT image artifacts and existence of small high‐density objects

**FIGURE 6:**
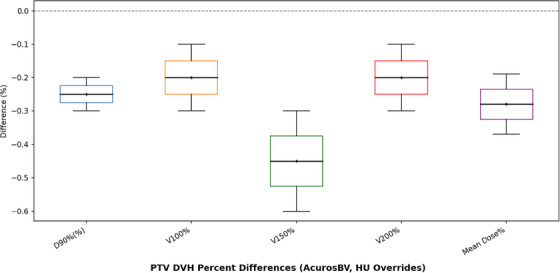
Box‐and‐whisker plot of DVH metrics, calculated by the AcurosBV algorithm, in PTV illustrating the percentage differences with and without HU overrides of artifacts and high‐density objects.

A subset of three plans containing BB markers, dummy sources, and visible imaging artifacts were reprocessed for evaluation of their impact on dose calculation. These elements were contoured and assigned HU overrides with air for metallic BBs and dummies, and water or air for artifacts depending on locations before recalculating dose using AcurosBV. The average total volume of all override structures is 70.83 ± 18.54 cc. The recalculated plans exhibited negligible changes in DVH metrics, with the most significant differences in PTV ranging from 0% to 0.6% as shown in Figure 6. These results indicate that the presence of minor artifacts and small high‐density objects may not significantly influence the plan evaluation with the DVH metrics used in this study.

## 4 DISCUSSION

This study provides a targeted comparison between TG‐43 and the HU‐based AcurosBV algorithm for HDR brachytherapy delivered with H.A.M. applicators in superficial head‐and‐neck sites. Across our cohort (17 nasal and 3 non‐nasal lesions), AcurosBV produced systematically lower values for PTV mean dose, D90%, and V100% compared with TG‐43. This behavior is expected because TG‐43 assumes an unbounded homogeneous water medium, whereas H.A.M. treatments, particularly in the nasal region, are strongly influenced by change of scatter at curved surfaces, adjacent air cavities, and bony structures. Accordingly, the average reduction in PTV V100% (approximately 6%) is consistent with heterogeneity and finite‐scatter effects that TG‐43 does not model.^7^, ^11^, ^14^ Because this is a small, single‐institution cohort dominated by nasal lesions, generalization to other superficial head‐and‐neck subsites should be made cautiously.^4^

Prior TG‐43 versus MBDCAs comparisons in other HDR brachytherapy settings (e.g., breast APBI and gynecologic/cervix) similarly report that TG‐43 can overestimate target coverage and hotspot metrics when heterogeneities, shielding, or bounded geometries reduce scatter.^22^, ^23^, ^24^, ^25^ Monte Carlo and deterministic radiation transport algorithm benchmarking studies further support the physical basis of model‐based calculations and provide commissioning frameworks for bounded homogeneous geometries.^26^, ^27^ Recent superficial mold studies of extensive scalp lesions similarly report clinically relevant TG‐43 overestimation relative to model‐based/Monte Carlo approaches, supporting adoption of MBDCAs for broad superficial targets.^19^

For clarity, plans were prescribed 5 Gy per fraction to 3–5 mm under the skin surface. The TG‐43 versus AcurosBV comparison was performed on a per‐patient basis (one plan per patient, *n* = 20); AcurosBV was used as a retrospective recalculation, thereby isolating algorithmic effects. For context, 40 Gy in 8 fractions of 5 Gy corresponds to EQD2 approximately 50 Gy for tumor tissue (alpha/beta = 10 Gy) and EQD2 approximately 64 Gy for late‐responding normal tissue (alpha/beta = 3 Gy).^28^ Outcome‐based evidence linking an AcurosBV‐derived reduction in V100% to tumor control probability for H.A.M. nasal or superficial head‐and‐neck lesions remains limited; therefore, the observed V100% reduction should be interpreted cautiously when translating TG‐43‐based coverage expectations to model‐based dose calculations.

The skin demonstrated the largest algorithmic difference (approximately 4%–5% reduction in D0.1cc–D2cc with AcurosBV). In superficial brachytherapy, small‐volume high‐dose metrics (e.g., D0.1cc and D0.2cc) are commonly used as surrogates for peak skin dose and are emphasized in consensus guidance and reviews of skin brachytherapy practice.^29^, ^30^ Our findings therefore suggest that algorithm choice can meaningfully affect reported skin hotspots and should be considered when comparing plans against TG‐43‐derived constraints or when auditing toxicity. Notably, the skin showed the most substantial OAR dose differences between algorithms, with reductions of approximately 4%–5% for D0.1cc to D2cc observed with AcurosBV. This is consistent with the study from Howie et al., who reported on average a 2% difference between TG‐43 and model‐based dose calculations in 100 patients receiving interstitial partial breast irradiation skin dose.^31^ This may be explained by the irregularity and curvature of the treatment surface, such as the nose, where air gaps between the H.A.M applicator and skin may exist. These geometric challenges introduce additional scattering complexity not captured by TG‐43 formalism. In contrast, other OARs such as the optical nerves, lenses, and brain showed minimal differences, likely due to their greater distance from the treatment volume and their relatively low doses. The statistical significance of even small differences in these structures suggests that while the magnitude may be limited, the MBDCA offers a more anatomically realistic representation of dose distribution.

This study did not directly measure applicator–skin air gaps or correlate CT‐derived gap thickness with dosimetric differences, which would strengthen mechanistic interpretation, particularly for nasal cases where air cavities and curvature are prominent. In addition, independent third‐party verification (e.g., Monte Carlo or film/phantom measurements) was not performed in this retrospective dataset; prior validation and commissioning studies for model‐based dose calculations provide useful frameworks for such verification.^9^, ^10^, ^31^, ^32^ Future work should incorporate quantitative gap assessment, stratify results by anatomical subsite, and include independent verification where feasible to quantify absolute deviations of each algorithm.

Our artifact sensitivity analysis included three cases selected a priori as worst‐case scenarios because they contained prominent CT streaking artifacts and/or BB markers and dummy wires within or near the PTV. HU override had negligible impact (<0.6% in PTV DVH metrics), suggesting limited sensitivity of AcurosBV to these small high‐density structures for the metrics evaluated here. However, this subset remains small; broader evaluation is warranted before generalizing to other artifact types or larger high‐Z materials. More generally, HU override can introduce uncertainty when large volumes are reassigned or when the override material choice is ambiguous^7^; in this study, override volumes were small, and the sensitivity analysis suggests minimal bias for the DVH metrics reported.

From a clinical implementation perspective, these data support a staged, practical transition to MBDCAs for H.A.M. applicator planning. A feasible approach includes: (1) dual reporting of TG‐43 and AcurosBV during an implementation period; (2) development of site‐specific guidance or conversion factors for key metrics (e.g., PTV D90 and skin D0.1cc/D0.2cc); (3) re‐evaluation of institutional dose constraints using AcurosBV‐recalculated historical plans; and (4) commissioning and periodic QA consistent with TG‐186 recommendations. Finally, as emphasized in TG‐186, caution in constraint translation applies broadly to model‐based dose calculation algorithms and is not specific to AcurosBV alone.^7^, ^33^

## 5 CONCLUSION

This study demonstrates that the HU‐based AcurosBV algorithm consistently yields lower and more anatomically informed dose estimates than TG‐43 for HDR brachytherapy using H.A.M. applicators in superficial head‐and‐neck sites. The most notable differences were observed in target coverage (PTV D90% and V100%) and in skin hotspot metrics (D0.1cc‐D2cc), reflecting sensitivity to finite‐scatter conditions, air cavities, and surface curvature that are not represented in TG‐43.

The observed differences represent the clinically relevant reporting changes that could affect plan evaluation against TG‐43‐based historical constraints. While AcurosBV is expected to be more physically realistic in heterogeneous, near‐surface geometries, direct clinical substitution should be accompanied by local validation and reassessment of dose constraints. A staged transition with dual reporting, institutional constraint translation, and outcome‐informed refinement is a practical interim strategy.

## AUTHOR CONTRIBUTIONS

**Pelei E. Kpohou**: Data curation; Formal analysis; Investigation; Visualization; Writing—original draft; Writing—review & editing. **Lyu Huang**: Conceptualization; Methodology; Validation; Project administration; Supervision; Writing—review & editing. **Lin Wang**: Resources; Methodology; Validation; Writing—review & editing. **Michael Trager**: Methodology; Validation; Writing—review & editing. **Yijian Cao**: Conceptualization; Resources; Supervision; Validation; Writing—review & editing.

## CONFLICT OF INTEREST STATEMENT

The authors declare no conflicts of interest related to this work.

## Data Availability

The data that support the findings of this study are available from the corresponding author upon reasonable request.
